# Associations of adverse childhood experiences and social support with self-injurious behaviour and suicidality in adolescents

**DOI:** 10.1192/bjp.2018.263

**Published:** 2019-03

**Authors:** Yuhui Wan, Ruoling Chen, Shuangshuang Ma, Danielle McFeeters, Ying Sun, Jiahu Hao, Fangbiao Tao

**Affiliations:** 1 Associated Professor, Department of Maternal, Child and Adolescent Health, School of Public Health, Anhui Medical University, China, Anhui Provincial Key Laboratory of Population Health and Aristogenics, Anhui Medical University, China and Faculty of Education, Health and Wellbeing, University of Wolverhampton, UK; 2 Professor, Faculty of Education, Health and Wellbeing, University of Wolverhampton, UK; 3 Student, Department of Maternal, Child and Adolescent Health, School of Public Health, Anhui Medical University and Anhui Provincial Key Laboratory of Population Health and Aristogenics, Anhui Medical University, China; 4 Fellow, Faculty of Education, Health and Wellbeing, University of Wolverhampton, UK; 5 Associated Professor, Department of Maternal, Child and Adolescent Health, School of Public Health, Anhui Medical University and Anhui Provincial Key Laboratory of Population Health and Aristogenics, Anhui Medical University, China; 6 Professor, Department of Maternal, Child and Adolescent Health, School of Public Health, Anhui Medical University and Anhui Provincial Key Laboratory of Population Health and Aristogenics, Anhui Medical University, China; 7 Professor, Department of Maternal, Child and Adolescent Health, School of Public Health, Anhui Medical University and Anhui Provincial Key Laboratory of Population Health and Aristogenics, Anhui Medical University, China

**Keywords:** Adverse childhood experiences, social support, non-suicidal self-injury, suicidal ideation, suicide attempt

## Abstract

**Background:**

There is little investigation on the interaction effects of adverse childhood experiences (ACEs) and social support on non-suicidal self-injury (NSSI), suicidal ideation and suicide attempt in community adolescent populations, or gender differences in these effects.

**Aims:**

To examine the individual and interaction effects of ACEs and social support on NSSI, suicidal ideation and suicide attempt in adolescents, and explore gender differences.

**Method:**

A school-based health survey was conducted in three provinces in China between 2013–2014. A total of 14 820 students aged 10–20 years completed standard questionnaires, to record details of ACEs, social support, NSSI, suicidal ideation and suicide attempt.

**Results:**

Of included participants, 89.4% reported one or more category of ACEs. The 12-month prevalence of NSSI, suicidal ideation and suicide attempt was 26.1%, 17.5% and 4.4%, respectively; all were significantly associated with increased ACEs and lower social support. The multiple adjusted odds ratio of NSSI in low versus high social support was 2.27 (95% CI 1.85–2.67) for girls and 1.81 (95% CI 1.53–2.14) for boys, and their ratio (Ratio of two odds ratios, ROR) was 1.25 (*P* = 0.037). Girls with high ACEs scores (5–6) and moderate or low social support also had a higher risk of suicide attempt than boys (RORs: 2.34, 1.84 and 2.02, respectively; all *P* < 0.05).

**Conclusions:**

ACEs and low social support are associated with increased risk of NSSI and suicidality in Chinese adolescents. Strategies to improve social support, particularly among female adolescents with a high number of ACEs, should be an integral component of targeted mental health interventions.

**Declaration of interest:**

None.

Non-suicidal self-injury (NSSI), suicidal ideation and suicide attempt are major public health problems in adolescents worldwide,^1^^,^^2^ and they represent some of the strongest and most consistent predictors of future suicidal behaviour across both in-patient and general populations.^1^^,^^3^ To sustain improvements in management and prevention initiatives, research continues to strive to better comprehend the complex interplay between many of the recognised psychosocial risk factors. Thus far, a substantial body of research has demonstrated significant independent effects between adverse childhood experiences (ACEs) and social support on self-injurious behaviour (SIB) and suicidality.^4^^,^^5^ Yet current knowledge surrounding these relationships is predominantly derived from Western countries and from adult or clinical populations,^4^^,^^5^ with only a few studies undertaken in community adolescent populations in China.^2^^,^^6^ There is also little research on the interaction effects between ACEs and social support on NSSI, suicidal ideation and suicide attempt in adolescents, despite ACEs and social support being highly correlated.^7^ Finally, despite evidence differentiating boys and girls in terms of the prevalence and effects of different ACEs,^8^ the perceptions and utilisation of social support^9^ and the presentation of NSSI, suicidal ideation and suicide attempt,^1^ few studies have been undertaken to examine gender differences in the interaction between ACEs and social support on NSSI, suicidal ideation and suicide attempt. This is particularly important in non-Western populations where there is a dearth of research despite the cultural context in China, which continues to demonstrate inherent gender discrimination.^10^^,^^11^ Therefore, our study first sought to investigate the independent effects of ACEs and social support on NSSI, suicidal ideation and suicide attempt in Chinese community adolescents; second, sought to examine the interaction effects between ACEs and social support on NSSI, suicidal ideation and suicide attempt and third, sought to ascertain whether there are any apparent gender differences in either independent or interaction effects for these relationships.

## Method

### Study sample and procedures

Three provinces, namely Anhui, Henan and Guizhou, were chosen as our study fields for data collection. These provinces are broadly representative of the average population within China in terms of economic development and demographic composition, and are also where our adolescent health research network is located, thus facilitating the data collection. In each province, one region (Bengbu in Anhui province, Zhengzhou in Henan province and Guiyang in Guizhou province) was randomly selected. In each region, eight general junior and senior schools (four from rural areas) were randomly chosen to recruit participants. As 4 schools were combined junior and senior schools, only 20 schools were selected for inclusion in the survey. In total, 15 278 students aged 10–20 years, from grades 7–12, were contacted for this health survey and asked to complete an anonymous questionnaire. Informed consent was sought from parents/guardians, and 1.5% of the recruited participants or their parents/guardians opted out of the study. The design and data collection procedures were approved by the Ethics Committee of Anhui Medical University (2012534). The survey was conducted from November 2013 to January 2014.

### Measurement of sociodemographic profile, psychological symptoms, ACEs and social support

#### Sociodemographic profile and psychological symptoms

Demographic data for each participant was recorded, including age, gender (boys or girls), urban/rural residency, parents' education level (less than junior middle school, junior middle school, senior middle school, college or more) and self-perceived economic status of the family (poor, moderate or good). Psychological symptoms, including emotional, behavioural and social adaptation symptoms, were evaluated by the psychological domain of the Multidimensional Sub-health Questionnaire of Adolescents^12^ (Cronbach's *α* = 0.920 in this study).

#### ACEs

ACEs were defined as having experienced childhood maltreatment and/or household dysfunction. Childhood maltreatment was evaluated by the Child Trauma Questionnaire (CTQ),^13^ a widely used 28-item measure that assesses 5 different forms of childhood trauma (physical abuse, sexual abuse, emotional abuse, physical neglect and emotional neglect). The CTQ was translated and validated in Chinese.^14^ The participants were asked about abusive childhood experiences before 16 years of age. Responses ranged from ‘never true’ to ‘rarely true’, ‘sometimes true’, ‘often true’ or ‘very often true’. Respondents were defined as exposed to a category if they responded ‘very often’, ‘often’ or ‘sometimes’ to any item in that category. A Cronbach's *α* coefficient of 0.737 was obtained for the CTQ in the current study. Household dysfunction questions were derived from the Centers for Disease Control and Kaiser Permanente Adverse Childhood Experiences Study in the USA.^8^ Household dysfunction was assessed through endorsement of the following experiences: (a) the divorce/separation of parents, (b) a parent serving time in jail, (c) having witnessed domestic violence, (d) having lived with someone who was mentally ill or suicidal or (e) having lived with someone with an alcohol or drug problem. Respondents were classified as exposed to household dysfunction if they responded ;yes; to any item. The Cronbach's *α* coefficient for the household dysfunction questionnaire was 0.705 in the present study. Because of the high interrelatedness of various types of ACEs (all *P* < 0.01), an ordinal number of ACEs categories score was created by summing the dichotomous ACEs items (range, 0 (unexposed) to 6 (exposed to physical abuse, sexual abuse, emotional abuse, physical neglect, emotional neglect and household dysfunction)) to investigate the graded association of ACEs and both SIB and suicidality.^8^ The total score was then converted into four categories of summed score (0, 1–2, 3–4 and 5–6), with zero experiences selected as the referent for analysis purposes.

#### Social support

Social support was assessed by the 17-item Adolescent Social Support Scale,^15^ which includes three dimensions: objective support, subjective support and support availability. Participants reported whether an item from the scale was in ‘inconformity’, ‘little inconformity’, ‘uncertainty’, ‘little conformity’ or ‘conformity’. The scale scores, with a possible range of 17–85 (low to high social support), had a good internal consistency in the present study, with a significant Cronbach's *α* coefficient of 0.940. The total score was divided into three levels (high, *P*_75_–*P*_100_; moderate, *P*_25_–*P*_75_; and low, *P*_0_–*P*_25_) for analysis.

### Measurements of NSSI, suicidal ideation and suicide attempt

#### NSSI

All participants received a screening questionnaire for NSSI, asking ‘In the past 12 months, have you ever harmed yourself in a way that was deliberate, but not intended to take your life?’. A list of eight NSSI methods were specified: hit yourself, pulled your own hair, banged your head or fist against something, pinched or scratched yourself, bitten yourself, cut or pierced yourself and burned yourself. Participants were then asked, ‘Have you ever done something with the intention of hurting yourself other than what was presented?’.^6^ For those who confirmed that they had engaged in NSSI, the frequency of NSSI was investigated. NSSI was dichotomised (frequency of NSSI of three or more versus fewer than three as yes or no, respectively) for analysis. The internal consistency reliability of NSSI was 0.749 in the current study.

#### Suicidal ideation and suicide attempt

Suicidal ideation and suicide attempt refer to the ‘middle school questionnaire’ of the 2013 Youth Risk Behaviour Surveillance System in the USA.^16^ Suicidal ideation was defined as a ‘yes’ in response to the question ‘Have you ever thought about killing yourself in the past 12 months?’. Suicide attempt was defined as a ‘yes’ in response to the question ‘Have you ever tried to kill yourself in the past 12 months?’.

### Statistical analysis

Of the 15 278 school adolescents recruited, 458 (3.0%) were excluded from the study because of absence from school on the day of the survey or unwillingness to respond to the questionnaire, or high levels of missing data or obviously fictitious or inconsistent responses. Thus, a total sample of 14 820 (97.0%) participants was analysed.

Sociodemographic data, ACEs, social support, NSSI, suicidal ideation and suicide attempt were described in both the total population and for boys and girls separately. Gender differences were assessed with the χ^2^-test for categorical variables and one-way analysis of variance for continuous variables. Binomial logistic regression models were used to examine the associations of NSSI, suicidal ideation and suicide attempt with ACEs and social support individually, and then in combination. In the models, adjustment was made for age, gender, regional area, school, urban/rurality, mother's education level, economic status of family and psychological symptoms. In examining the association of NSSI with ACEs and social support, we also used the thresholds of NSSI score  **≥**1 and **≥**5 for sensitivity analysis.

Gender differences in the associations were examined via two odds ratios (Ratio of two odds ratios, RORs).^17^ All analyses were conducted with SPSS software, Windows version 16.0 (SPSS Inc., Chicago, IL).

## Results

### Characteristics of participants

Of the 14 820 participants, the mean age was 15.4 years (s.d. 1.8), and 50.2% were girls. Scores showed that 45.7% of the sample had experienced childhood emotional abuse, 20.3% had experienced physical abuse, 13.3% had experienced sexual abuse, 64.2% had experienced physical neglect, 58.5% had experienced emotional neglect and 42.5% had experienced household dysfunction; in total, 89.4% had experienced one or more ACEs and 46.3% reported three or more types of ACEs. Compared with boys, girls had significantly more psychological symptoms, fewer ACEs and a higher level of social support (*P* < 0.001). Boys had significantly increased exposure to physical and sexual abuse, physical and emotional neglect, household dysfunction and NSSI (*P* < 0.001). Girls had significantly greater exposure to emotional abuse, suicidal ideation and suicide attempt (*P* < 0.001). The details of gender differences and sociodemographic factors can be seen in Table 1.

**Table 1:** Characteristics of participants by gender, data shown as *n* (%)

Variables	Total	Girl	Boy	*P*-value
Age (mean, s.d.)	15.44 (1.8)	15.46 (1.8)	15.41 (1.8)	0.112
Regional areas				
Zhengzhou	5087 (34.3)	2605 (35.0)	2482 (33.6)	<0.001
Guiyang	4617 (31.2)	2499 (33.6)	2118 (28.7)	
Bengbu	5116 (34.5)	2339 (31.4)	2777 (37.6)	
Urban/rurality				
Urban	6125 (41.3)	3080 (41.4)	3045 (41.3)	0.897
Rural	8695 (58.7)	4363 (58.6)	4332 (58.7)	
Father's education level				
College or more	2230 (15.0)	1129 (15.2)	1101 (14.9)	0.012
Senior middle school	3129 (21.1)	1529 (20.5)	1600 (21.7)	
Junior middle school	6039 (40.7)	2988 (40.1)	3051 (41.4)	
Less than junior middle school	3422 (23.1)	1797 (24.1)	1625 (22.0)	
Mother's education level				
College or more	1706 (11.5)	835 (11.2)	871 (11.8)	0.277
Senior middle school	2826 (19.1)	1413 (19.0)	1413 (19.2)	
Junior middle school	5369 (36.2)	2672 (35.9)	2697 (36.6)	
Less than junior middle school	4919 (33.2)	2523 (33.9)	2396 (32.5)	
Economic status of family				
Good	1841 (12.4)	845 (11.4)	996 (13.5)	<0.001
Moderate	10 306 (69.6)	5394 (72.5)	4912 (66.6)	
Poor	2673 (18.0)	1204 (16.2)	1469 (19.9)	
Psychological symptoms				
Yes	3572 (24.1)	1932 (26.0)	1640 (22.2)	<0.001
No	11 248 (75.9)	5511 (74.0)	5737 (77.8)	
Adverse childhood experiences score				
0	1573 (10.6)	882 (11.9)	691 (9.4)	<0.001
1–2	6388 (43.1)	3316 (44.6)	3072 (41.6)	
3–4	5303 (35.8)	2602 (35.0)	2701 (36.6)	
5–6	1556 (10.5)	643 (8.6)	913 (12.4)	
Emotional abuse	6770 (45.7)	3664 (49.2)	3106 (42.1)	<0.001
Physical abuse	3008 (20.3)	1245 (16.7)	1763 (23.9)	<0.001
Sexual abuse	1969 (13.3)	709 (9.5)	1260 (17.1)	<0.001
Emotional neglect	9515 (64.2)	4599 (61.8)	4916 (66.6)	<0.001
Physical neglect	8670 (58.5)	4061 (54.6)	4609 (62.5)	<0.001
Household dysfunction	6303 (42.5)	3101 (41.7)	3202 (43.4)	0.032
Level of social support				
High	3635 (24.5)	1934 (26.0)	1701 (23.1)	<0.001
Moderate	7272 (49.1)	3816 (51.3)	3456 (46.8)	
Low	3913 (26.4)	1693 (22.7)	2220 (30.1)	
Non-suicidal self-injury	3872 (26.1)	1811 (24.3)	2061 (27.9)	<0.001
Suicide ideation	2588 (17.5)	1465 (19.7)	1123 (15.2)	<0.001
Suicide attempt	649 (4.4)	390 (5.2)	259 (3.5)	<0.001

### Effect of ACEs and social support on NSSI, suicidal ideation and suicide attempt, and gender difference

Table 2 shows the number and percentage of participants according to NSSI category, among different levels of ACEs and social support. There were significant trends toward increased NSSI with higher ACEs and lower social support. Multiple adjusted odds ratios for NSSI were significantly increased with higher ACEs and lower social support (model 2 in Table 2). Even when ACEs and social support were included in the model simultaneously, there were main effects of ACEs score and level of social support on NSSI (model 3 in Table 2). Using the thresholds of NSSI ≥ 1 and NSSI ≥ 5 for separate data analysis, we found that the associations of ACEs score and level of social support (Supplementary Table 1 available at https://doi.org/10.1192/bjp.2018.263) were similar to those found with NSSI ≥ 3 (Table 2).

**Table 2:** Number, per cent and odds ratio of NSSI by ACEs score and level of social support

Variables	*n* (%)		Model 1^a^			Model 2^b^			Model 3^b^		
	NSSI < 3	NSSI ≥ 3	Odds ratio	95%CI	*P*-value	Odds ratio	95%CI	*P*-value	Odds ratio	95%CI	*P*-value
ACEs											
0	1404 (89.3)	169 (10.7)	1.0			1.0			1.0		
1–2	5078 (79.5)	1310 (20.5)	2.14	1.81–2.54	<0.001	1.98	1.66–2.36	<0.001	1.86	1.56–2.23	<0.001
3–4	3624 (68.3)	1679 (31.7)	3.85	3.25–4.56	<0.001	3.17	2.65–3.79	<0.001	2.80	2.34–3.35	<0.001
5–6	842 (54.1)	714 (45.9)	7.05	5.84–8.50	<0.001	5.39	4.40–6.60	<0.001	4.65	3.79–5.70	<0.001
Social support											
High	3105 (85.4)	530 (14.6)	1.0			1.0			1.0		
Moderate	5407 (74.4)	1865 (25.6)	2.02	1.82–2.25	<0.001	1.75	1.56–1.95	<0.001	1.61	1.43–1.80	<0.001
Low	2436 (62.3)	1477 (37.7)	3.55	3.17–3.98	<0.001	2.42	2.14–2.73	<0.001	2.00	1.77–2.27	<0.001

ACEs, adverse childhood experiences; NSSI, non-suicidal self-injury.

a. Unadjusted model.

b. Adjusted for gender, age, regional areas, school, urban/rurality, mother's education level, economic status of family and psychological symptoms.

c. Adjusted for gender, age, regional areas, school, urban/rurality, mother's education level, economic status of family, psychological symptoms, ACEs score and social support level.

Supplementary Table 2 shows data on suicidal ideation and suicide attempt in relation to ACEs and social support. There were significant trends toward increased suicidal ideation and suicide attempt with higher ACEs and lower social support. Multiple adjusted odds ratios for suicidal ideation and suicide attempt were significantly increased with higher ACEs and lower social support, respectively. When ACEs and social support were put in the model simultaneously, the main effects of the ACEs and social support on suicidal ideation and suicide attempt remained, with the exception of social support on suicide attempt in boys.

No gender differences were found in the independent effects of ACEs or social support on NSSI, with the exception of the lowest social support having a stronger effect in girls than in boys (Table 3). In the data analysis for NSSI ≥ 1 and NSSI ≥ 5, gender differences in ACEs score or social support on NSSI (Supplementary Tables 3 and 4) were similar to those found with NSSI ≥ 3 (Table 3), whereas the NSSI ≥ 5 data showed a borderline significance for the lowest social support having a stronger effect in girls than in boys. There were no gender differences in the effects of ACEs or social support on suicidal ideation (Supplementary Table 5), but the effects of high ACEs score and low or moderate social support on suicide attempt were significantly stronger in girls than in boys (Table 4).

**Table 3:** Number, per cent and odds ratio of NSSI by ACEs score and level of social support in girls and boys, and the gender ratio

Variables	Girls				Boys				Ratio of two odds ratios in girls versus boys^b^	
	*n* (%)	Odds ratio (95% CI)^a^	Odds ratio (95% CI)^b^	*P*-value^b^	*n* (%)	Odds ratio (95% CI)^a^	Odds ratio (95% CI)^b^	*P*-value^b^	Ratio of two odds ratios (ROR)	One-sided *P*-value
ACEs										
0	81 (9.2)	1.0	1.0		88 (12.7)	1.0	1.0			
1–2	631 (19.0)	2.32 (1.82–2.97)	1.92 (1.49–2.47)	<0.001	679 (22.1)	1.94 (1.53–2.47)	1.80 (1.40–2.31)	<0.001	1.07	0.361
3–4	803 (30.9)	4.41 (3.46–5.63)	2.89 (2.27–3.74)	<0.001	876 (32.4)	3.29 (2.59–4.17)	2.69 (2.09–3.47)	<0.001	1.07	0.346
5–6	296 (46.0)	8.44 (6.40–11.12)	5.13 (3.80–6.93)	<0.001	418 (45.8)	5.79 (4.47–7.50)	4.30 (3.24–5.70)	<0.001	1.19	0.201
Social support										
High	244 (12.6)	1.0	1.0		286 (16.8)	1.0	1.0			
Moderate	922 (24.2)	2.21 (1.89–2.57)	1.68 (1.43–1.98)	<0.001	943 (27.3)	1.86 (1.60–2.15)	1.53 (1.31–1.79)	<0.001	1.10	0.208
Low	832 (37.5)	4.27 (3.61–5.03)	2.27 (1.85–2.67)	<0.001	645 (38.1)	2.97 (2.54–3.46)	1.81 (1.53–2.14)	<0.001	1.25	0.037

ACEs, adverse childhood experiences; NSSI, non-suicidal self-injury.

a. Unadjusted model.

b. Adjusted for age, regional areas, school, urban/rurality, mother's education level, economic status of family, psychological symptoms, ACEs score and level of social support.

c. Calculated by adjusted odds ratio.

**Table 4:** Number, per cent and odds ratio of suicide attempt by ACEs score and level of social support in girls and boys, and the gender ratio

Variables	Girls				Boys				Ratio of two odds ratios in girls versus boys^c^	
	*n* (%)	Odds ratio (95% CI)^a^	Odds ratio (95% CI)^b^	*P*-value^b^	*n* (%)	Odds ratio (95% CI)^a^	Odds ratio (95% CI)^b^	*P*-value^b^	Ratio of two odds ratios (ROR)	One-sided *P*-value
ACEs										
0	14 (1.6)	1.0	1.0		12 (1.7)	1.0	1.0			
1–2	103 (3.1)	1.99 (1.13–3.49)	1.68 (0.95–2.98)	0.075	60 (2.0)	1.13 (0.60–2.11)	1.00 (0.53–1.89)	0.998	1.68	0.117
3–4	172 (6.6)	4.39 (2.53–7.61)	3.07 (1.74–5.43)	<0.001	120 (4.4)	2.63 (1.45–4.79)	2.04 (1.10–3.80)	0.025	1.50	0.171
5–6	101 (15.7)	11.56 (6.54–20.41)	7.14 (3.92–13.01)	<0.001	67 (7.3)	4.48 (2.40–8.35)	3.05 (1.57–5.92)	0.001	2.34	0.031
Social support										
High	51 (2.6)	1.0	1.0		50 (2.9)	1.0	1.0			
Moderate	158 (4.1)	1.60 (1.16–2.20)	1.40 (1.00–1.97)	0.048	82 (2.4)	0.80 (0.56–1.15)	0.76 (0.53–1.11)	0.158	1.84	0.008
Low	181 (10.7)	4.42 (3.22–6.08)	2.62 (1.85–3.71)	<0.001	127 (5.7)	2.00 (1.44–2.80)	1.30 (0.90–1.87)	0.165	2.02	0.003

ACEs, Adverse childhood experiences; NSSI, non-suicidal self-injury.

a. Unadjusted model.

b. Adjusted for age, regional areas, school, urban/rurality, mother's education level, economic status of family, psychological symptoms, ACEs score and level of social support.

c. Calculated by adjusted odds ratio.

Further analysis was conducted to examine whether specific types of ACEs demonstrated gender differences in the effect on NSSI, suicidal ideation and suicide attempt (Supplementary Tables 6–8). Each type of ACEs was significantly associated with NSSI, suicidal ideation and suicide attempt in boys and girls, except for emotional and physical neglect with suicide attempt in boys (Supplementary Table 8). Emotional abuse increased the risk of NSSI, suicidal ideation and suicide attempt more than other types of ACEs among both genders. When exposed to emotional abuse, girls were more likely to engage in NSSI than boys (Supplementary Table 6). When exposed to physical abuse, girls were also more likely to report a suicide attempt than boys (Supplementary Table 8). Conversely, suicidal ideation was more common among boys than girls when exposed to emotional neglect (Supplementary Table 7). No further gender differences were found.

### Interaction effect between ACEs and social support on NSSI, suicidal ideation and suicide attempt, and gender difference

Although ACEs and social support were highly correlated in the present study (Supplementary Table 9), there were no interaction effects between ACEs and social support on NSSI (total sample, *P* = 0.062; boys, *P* = 0.521; girls, *P* = 0.115), and suicidal ideation (total sample, *P* = 0.087; boys, *P* = 0.061; girls, *P* = 0.075). However, there was an interaction effect of ACEs and social support on suicide attempt in the total sample (*P* = 0.001) and in boys (*P* = 0.002), although it was not significant in girls (*P* = 0.334).

## Discussion

A large-scale, school-based survey was conducted to examine the independent and interaction effects between ACEs and social support on NSSI, suicidal ideation and suicide attempt in adolescents. Our data reveals that ACEs and social support have main effects on both SIB and suicidality independently, as well as interaction effects between ACEs and social support on suicide attempt but not NSSI or suicidal ideation. Similar relationships are found across genders; however, girls were more likely to engage in NSSI and suicide attempt when social support was low, and an interaction effect between ACEs and social support on suicide attempt was found only in boys.

### The effects of ACEs on NSSI, suicidal ideation and suicide attempt

Previous research has shown a relationship between ACEs, NSSI and suicidality.^4^ An investigation in 957 undergraduate students in Canada suggested that those experiencing more adverse family-life events and higher perceived relational trauma were more at risk of engaging in NSSI behaviours.^18^ A prospective study in youths aged 14–26 years also suggested that physical abuse, emotional abuse and emotional neglect were associated with subsequent risk of suicidal behaviour.^19^ Our study has extended this literature by demonstrating gender-specificity effects in the relationships, and by addressing the limitation of the lack of equivalent research within Chinese community populations. However, the relationships observed between all maltreatment types and NSSI, suicidal ideation and suicide attempt conflicts with other studies^20^ in clinically referred youth, which indicate that only indirect childhood maltreatment (i.e. witnessing domestic violence) is significantly associated with NSSI, whereas direct forms of maltreatment (physical or sexual abuse) are not. Similarly in adult samples, varied effects were found with different forms of childhood adversity, as childhood abuse was not significantly associated with NSSI and suicide attempt after adjusting for the correlation with low maternal or paternal care.^21^ Thus, various study samples, different definitions of ACEs and diverse control variables should be considered to interpret the results of the study.

The tradition of son preference remains prevalent in China.^10^ This may contribute, at least in part, to the poorer mental health outcomes (including depression, anxiety, low self-esteem, sensitivity to negative life events and interpersonal pressure) previously observed in female Chinese school children.^22^ Yet, no study has attempted to identify gender differences in the relationships between ACEs, SIB and suicidality in Chinese adolescents. Although the risk of NSSI, suicidal ideation and suicide attempt increased in line with a greater number of ACEs categories generally, our study suggests that girls who experienced a higher number of ACEs (categories) seem to be more vulnerable to suicide attempt (but not NSSI or suicidal ideation) than boys, which is in line with prior studies.^23^^,^^24^ Moreover, girls were found to be more susceptible to NSSI and suicide attempt when they encountered particular forms of maltreatment, primarily emotional or physical abuse. This substantiates findings from a series of studies, including Isohookana *et al*,^23^ who examined psychiatric in-patients aged 12–17 years and found that a higher number of ACEs was associated with an increased risk of NSSI and suicide attempt in girls, but not in boys. Garcia *et al*^24^ also showed that significant correlations were found between childhood trauma scores and psychotic symptoms, depressive symptoms and poorer functionality, but only in women, whereas childhood trauma was associated with poorer social cognition in both males and females. Further studies are needed to ascertain whether there are gender differences in the relationship between ACEs, SIB and suicidality.

### The effects of social support on NSSI, suicidal ideation and suicide attempt

Social support was found to have an independent effect on NSSI, suicidal ideation and suicide attempt, even after adjusting for sociodemographic risk factors, psychological symptoms and ACEs. Previous findings^5^^,^^25^ have also indicated that individuals with higher social support have a significantly lower odds of engaging in NSSI, suicidal ideation and suicide attempt, which is consistent with our study. One study in a clinical sample of adolescents suggested that perceptions of school support were independently and negatively associated with suicidal ideation, especially among adolescents who also reported perceptions of lower parent support.^5^ Furthermore, lower perceived parental support was independently associated with greater odds of history of suicide attempt. One study in community and in-patient mental health settings^25^ also showed that children and adolescents who had some form of social support had a 26% decrease in the odds of engaging in NSSI when compared with their counterparts who lacked social support. Collectively, this supports Ayub's^26^ suggestion that social support may play a significant role in the prevention of suicidal thoughts and behaviours, and that psychologists should include family and friends in their approaches to treating suicidal youth. This may be especially important in China, where mental illness is frequently blamed on the family and the individual.^11^ Moreover, considering that Chinese students are often burdened with tremendous academic pressure,^27^ support within the school environment may be particularly beneficial in this context.

The inverse association between social support and NSSI was found to be stronger in girls than in boys. This may contribute, in part, to the explanation of why the risk of suicidal behaviours is greater in girls despite having fewer ACEs and higher social support than boys. Traditionally within China, boys are more likely to be socialised to be independent than girls, which may help to account for the increased sensitivity to lower social support observed among females in our sample. Other studies examining developmental trajectories of suicidal ideation showed that support from family and friends differentiated suicidal ideation trajectories for both boys and girls.^28^ However, an ecological study in adults in 75 regions of 23 European countries revealed inverse relationships between social support and suicide rates for both genders, with some indication of a stronger relationship among men.^29^ Future studies may look to examine whether different types of social support (e.g. peer or parental support) are more influential for males or females.

### Interaction effects between ACEs and social support on NSSI, suicidal ideation and suicide attempt

Our study extends existing knowledge by demonstrating the interaction effect between ACEs and social support on suicide attempt, particularly in boys, but failed to establish a similar relationship for either NSSI or suicidal ideation. The relationship between ACEs, social support and SIBs and suicidality is complex. Christoffersen *et al*^7^ suggested that social support is a partial mediator between traumatic life-events and NSSI in young adulthood. A study of women in the USA found that the link between intimate partner violence and suicidal behaviour was moderated by social support.^30^ Extending this to the adolescent population, it would be reasonable to suggest that social support may also moderate the relationship between ACEs and SIBs in this population. That said, our study failed to establish interaction effects between ACEs and social support on NSSI and suicidal ideation. This may be partly attributable to the confounding effect of psychological symptoms, because these have found to be significantly related to ACEs, social support and SIB.^2^^,^^6^^,^^7^ In this study, statistical interaction effects of ACEs and social support on suicidal ideation were found after removing psychological symptoms from the multivariable model (results not shown). One possible alternative explanation may be that interaction effects are only apparent among those who are most seriously affected (such as those who have actual suicide attempts), as this group may be most likely to encounter the highest level of ACEs and lowest social support. Further studies on the interaction effects between ACEs and social support on NSSI, suicidal ideation and suicide attempt will be needed to further elucidate this complex interaction.

### Strengths and weakness of the study

This study is the first to examine gender differences and interaction effects between ACEs and social support on NSSI, suicidal ideation and suicide attempt within Chinese adolescents. The study sample is representative, covering urban and rural areas in China, and the response rate of the participants was high. The large sample from urban and rural areas provided enough statistical power to examine gender differences, with multivariate adjustment analysis. However, the study has limitations. First, the study was cross-sectional, therefore it is difficult to establish a causal relationship. Nonetheless, our findings pertaining to the association between ACEs and social support with SIB and suicidality were similar to those in previous cohort studies.^19^^,^^28^ Second, because of the use of self-reported questionnaires for data collection purposes, it is possible that recall bias may exist. This may ultimately influence the strength of the observed relationships, and our results may represent a more conservative estimation than is truly present. Third, the focus was solely directed toward the number and type of ACEs in this study, but it may be important to understand an individual's subjective experience of the events. Finally, the study focused on adolescents in traditional school environments, therefore the findings did not represent adolescents who were absent from school, which is important because studies have shown that ACEs, lower social support and suicidality are more prevalent in individuals with lower educational achievement and socioeconomic status.^31^ Caution should be exercised in the application of the findings to the whole population of adolescents in China.

### Implications

The findings indicate that ACEs and poorer social support are independently associated with an increased risk of both SIB and suicidality in school adolescents. Moreover, an interaction effect was observed between ACEs and social support on suicide attempt, which, by implication, suggests that the combination of these factors may be particularly detrimental in increasing the likelihood of behavioural enactment. In light of this, intervention and prevention strategies focused on enhancing perceived social support as a fundamental feature, particularly among female adolescents with a history of ACEs, may go some way toward mitigating the negative trajectory of ACEs in this population.

Educational settings are likely to represent an important conduit through which to improve the quality and accessibility of social support available to vulnerable adolescents. On a targeted level, awareness-raising initiatives should aim to integrate a training element specifically focused on the psychoeducation of teachers and other school staff. This should centre around both improving their recognition of the signs and symptoms of mental illness, particularly among individuals with a known history of ACEs, and on developing basic training in mental health literacy to facilitate appropriate emotional responses.^32^ On a universal school level, interpersonal skills training for pupils, provided in the context of an educational setting, may help to improve skills in social and peer interactions with a view to enhancing the quantity and quality of social support available to adolescents.^33^ Finally, as part of a broader systems approach, schools may also seek to improve perceptions of social support through increased participation in school and community life.^33^ This can be achieved through the strengthening of ties between schools and communities to facilitate greater cohesion and engagement in extracurricular activities, which will ultimately contribute to the construction of wider social networks for adolescents.

## Supplementary material

For supplementary material accompanying this paper visit https://doi.org/10.1192/bjp.2018.263. click here to view supplementary material

## References

[1] Hawton K, Saunders KE, O'Connor RC. Self-harm and suicide in adolescents. Lancet 2012; 379: 2373–82. 22726518 10.1016/S0140-6736(12)60322-5

[2] Tang J, Li G, Chen B, Huang Z, Zhang Y, Chang H, Prevalence of and risk factors for non-suicidal self-injury in rural China: results from a nationwide survey in China. J Affect Disord 2018; 226: 188–95. 28988001 10.1016/j.jad.2017.09.051

[3] Borowsky IW, Ireland M, Resnick MD. Adolescent suicide attempts: risks and protectors. Pediatrics 2001; 107: 485–93. 11230587 10.1542/peds.107.3.485

[4] Liu RT, Scopelliti KM, Pittman SK, Zamora AS. Childhood maltreatment and non-suicidal self-injury: a systematic review and meta-analysis. Lancet Psychiatry 2018; 5: 51–64. 29196062 10.1016/S2215-0366(17)30469-8 PMC5743605

[5] Miller AB, Esposito-Smythers C, Leichtweis RN. Role of social support in adolescent suicidal ideation and suicide attempts. J Adolesc Health 2015; 56: 286–92. 25561384 10.1016/j.jadohealth.2014.10.265

[6] Wan Y, Chen J, Sun Y, Tao F. Impact of childhood abuse on the risk of non-suicidal self-injury in mainland Chinese adolescents. PLoS ONE 2015; 10: e0131239. 26114574 10.1371/journal.pone.0131239 PMC4482708

[7] Christoffersen MN, Møhl B, DePanfilis D, Vammen KS. Non-Suicidal Self-Injury—Does social support make a difference? An epidemiological investigation of a Danish national sample. Child Abuse Negl 2015; 44: 106–16. 25435107 10.1016/j.chiabu.2014.10.023

[8] Felitti VJ, Anda RF, Nordenberg D, Williamson DF, Spitz AM, Edwards V, Relationship of childhood abuse and household dysfunction to many of the leading causes of death in adults: the Adverse Childhood Experiences (ACE) Study. Am J Prev Med 1998; 14: 245–58. 9635069 10.1016/s0749-3797(98)00017-8

[9] Rueger SY, Malecki CK, Demaray MK. Relationship between multiple sources of perceived social support and psychological and academic adjustment in early adolescence: comparisons across gender. J Youth Adolesc 2010; 39: 47–61. 20091216 10.1007/s10964-008-9368-6

[10] Hesketh T, Xing ZW. Abnormal sex ratios in human populations: causes and consequences. Proc Natl Acad Sci USA 2006; 103: 13271–5. 16938885 10.1073/pnas.0602203103 PMC1569153

[11] Zheng Y, Zheng X. Current state and recent developments of child psychiatry in China. Child Adolesc Psychiatry Ment Health 2015; 9: 10. 25972919 10.1186/s13034-015-0040-0 PMC4429456

[12] Tao F, Hu CL, Sun Y, Hao JH. The development and application of Multidimensional Sub-health Questionnaire of Adolescents (MSQA). Chin J Dis Control Prev 2008; 12: 309–14.

[13] Bernstein DP, Ahluvalia T, Pogge D, Handelsman L. Validity of the Childhood Trauma Questionnaire in an adolescent psychiatric population. J Am Acad Child Adolesc Psychiatry 1997; 36: 340–8. 9055514 10.1097/00004583-199703000-00012

[14] Zhao X, Zhang Y, Li L, Zhou Y, Li H, Yang S. Reliability and validity of the Chinese version of childhood trauma questionnaire. Chin J Clin Rehabil 2005; 9: 105–7.

[15] Ye Y, Dai X. Development of social support scale for university students. Chin J Clin Psychol 2008; 16: 456–8.

[16] Centers for Disease Control and Prevention (CDC). *Adolescent and School Health: YRBSS Questionnaire 2013.* CDC, 2013 (https://www.cdc.gov/healthyyouth/data/yrbs/questionnaires.htm).

[17] Altman DG, Bland JM. Interaction revisited: the difference between two estimates. BMJ 2003; 326: 219. 12543843 10.1136/bmj.326.7382.219 PMC1125071

[18] Martin J, Bureau J-F, Yurkowski K, Fournier TR, Lafontaine M-F, Cloutier P. Family-based risk factors for non-suicidal self-injury: considering influences of maltreatment, adverse family-life experiences, and parent–child relational risk. J Adolesc 2016; 49: 170–80. 27086083 10.1016/j.adolescence.2016.03.015

[19] Hadland SE, Wood E, Dong H, Marshall BD, Kerr T, Montaner JS, Suicide attempts and childhood maltreatment among street youth: a prospective cohort study. Pediatrics 2015; 136: 440–9. 26240210 10.1542/peds.2015-1108 PMC4552091

[20] Armiento J, Hamza CA, Stewart SL, Leschied A. Direct and indirect forms of childhood maltreatment and nonsuicidal self-injury among clinically-referred children and youth. J Affect Disord 2016; 200: 212–7. 27136420 10.1016/j.jad.2016.04.041

[21] Johnstone JM, Carter JD, Luty SE, Mulder RT, Frampton CM, Joyce PR. Childhood predictors of lifetime suicide attempts and non-suicidal self-injury in depressed adults. Aust N Z J Psychiatry 2016; 50: 135–44. 25999526 10.1177/0004867415585581

[22] Hou Z, Jia H, Guo J. A cross-sectional investigation of mental health level of 1397 middle school students. Med J Chin People's Health 2006; 18: 788–9.

[23] Isohookana R, Riala K, Hakko H, Räsänen P. Adverse childhood experiences and suicidal behavior of adolescent psychiatric inpatients. Eur Child Adolesc Psychiatry 2013; 22: 13–22. 22842795 10.1007/s00787-012-0311-8

[24] Garcia M, Montalvo I, Creus M, CabezasÁ SM, Algora MJ, Sex differences in the effect of childhood trauma on the clinical expression of early psychosis. Compr Psychiatry 2016; 68: 86–96. 27234188 10.1016/j.comppsych.2016.04.004

[25] Baiden P, Stewart SL, Fallon B. The role of adverse childhood experiences as determinants of non-suicidal self-injury among children and adolescents referred to community and inpatient mental health settings. Child Abuse Negl 2017; 69: 163–76. 28477476 10.1016/j.chiabu.2017.04.011

[26] Ayub N. Predicting suicide ideation through intrapersonal and interpersonal factors: the interplay of Big-Five personality traits and social support. Personal Ment Health 2015; 9: 308–18. 26148708 10.1002/pmh.1301

[27] Zhang Y, Deng G, Zhang Z, Zhou Q, Gao X, Di L, A cross sectional study between the prevalence of chronic pain and academic pressure in adolescents in China (Shanghai). BMC Musculoskelet Disord 2015; 16: 219. 26296558 10.1186/s12891-015-0625-z PMC4546215

[28] Adrian M, Miller AB, McCauley E, Vander Stoep A. Suicidal ideation in early to middle adolescence: sex-specific trajectories and predictors. J Child Psychol Psychiatry 2016; 57: 645–53. 26610726 10.1111/jcpp.12484 PMC4837032

[29] Šedivy NZ, Podlogar T, Kerr DC, De Leo D. Community social support as a protective factor against suicide: a gender-specific ecological study of 75 regions of 23 European countries. Health Place 2017; 48: 40–6. 28934635 10.1016/j.healthplace.2017.09.004

[30] Kaslow NJ, Thompson MP, Meadows LA, Jacobs D, Chance S, Gibb B, Factors that mediate and moderate the link between partner abuse and suicidal behavior in African American women. J Consult Clin Psychol 1998; 66: 533. 9642892 10.1037//0022-006x.66.3.533

[31] Kessler RC, Berglund P, Borges G, Nock M, Wang PS. Trends in suicide ideation, plans, gestures, and attempts in the United States, 1990–1992 to 2001–2003. JAMA 2005; 293: 2487–95. 15914749 10.1001/jama.293.20.2487

[32] Zalsman G, Hawton K, Wasserman D, van Heeringen K, Arensman E, Sarchiapone M, Suicide prevention strategies revisited: 10-year systematic review. Lancet Psychiatry 2016; 3: 646–59. 27289303 10.1016/S2215-0366(16)30030-X

[33] Ougrin D, Tranah T, Leigh E, Taylor L, Asarnow JR. Practitioner review: self-harm in adolescents. J Child Psychol Psychiatry 2012; 53: 337–50. 22329807 10.1111/j.1469-7610.2012.02525.x

