# Supplementary material for: Associations of adverse childhood experiences and social support with self-injurious behaviour and suicidality in adolescents
Source: Br J Psychiatry. 2019 Mar;214(3):146–52. doi: 10.1192/bjp.2018.263 (PMC6429251; doi:10.1192/bjp.2018.263)
Supplement: Supplementary file 1 [file S0007125018002635sup001.doc]

**Table DS1. Number, % and OR of NSSI defined as ≥1 and ≥5 by score of ACEs and level of SS: 20 schools study, China**

|  | **Threshold of NSSI ≥1 for analysis** | | | | | |  | **Threshold of NSSI ≥5 for analysis** | | | | | |
| --- | --- | --- | --- | --- | --- | --- | --- | --- | --- | --- | --- | --- | --- |
| Variable | N(%) | |  | Multi-variate adjusted analysis | | |  | N(%) | |  | Multi-variate adjusted analysis | | |
|  | NSSI=0 | NSSI**≥**1 |  | OR+ | 95%CI | *P*-Value |  | NSSI<5 | NSSI**≥**5 |  | OR+ | 95%CI | *P*-Value |
| **ACEs** |  |  |  |  |  |  |  |  |  |  |  |  |  |
| 0 | 1264(80.4) | 309(19.6) |  | 1.0 |  |  |  | 1458(92.7) | 115(7.3) |  | 1.0 |  |  |
| 1-2 | 4310(67.5) | 2078(32.5) |  | 1.77 | 1.53-2.03 | <.001 |  | 5467(85.6) | 921(14.4) |  | 1.85 | 1.50-2.28 | <.001 |
| 3-4 | 2884(54.4) | 2419(45.6) |  | 2.68 | 2.31-3.10 | <.001 |  | 4054(74.6) | 1249(23.6) |  | 2.80 | 2.27-3.47 | <.001 |
| 5-6 | 629(40.4) | 927(59.6) |  | 4.46 | 3.75-5.32 | <.001 |  | 985(63.3) | 571(36.7) |  | 4.72 | 3.73-5.97 | <.001 |
| **SS** |  |  |  |  |  |  |  |  |  |  |  |  |  |
| high | 2740(75.4) | 895(24.6) |  | 1.0 |  |  |  | 3269(89.9) | 366(10.1) |  | 1.0 |  |  |
| moderate | 4412(60.7) | 2860(39.3) |  | 1.58 | 1.44-1.74 | <.001 |  | 5943(81.7) | 1329(18.3) |  | 1.60 | 1.41-1.83 | <.001 |
| low | 1935(49.5) | 1978(50.5) |  | 1.88 | 1.69-2.10 | <.001 |  | 2752(70.3) | 1161(29.7) |  | 2.10 | 1.82-2.42 | <.001 |

Abbreviations: NSSI=non-suicidal self-injury; ACEs=Adverse childhood experiences; SS=social support

+Adjusted for gender, age, regional areas, school, urban/rurality, mother’s education level, economic status of family, psychological symptoms, ACEs and SS

**Table DS2. Number, % and OR of SI and SA by score of ACEs and level of SS: 20 schools study, China**

| Variables | No.(%) | |  | Model ^a^ | | |  | Model ^b^ | | |
| --- | --- | --- | --- | --- | --- | --- | --- | --- | --- | --- |
|  | No | Yes |  | OR | 95%CI | *P*-Value |  | OR | 95%CI | *P*-Value |
| **SI** |  |  |  |  |  |  |  |  |  |  |
| **ACEs** |  |  |  |  |  |  |  |  |  |  |
| 0 | 1462(92.9) | 111(7.1) |  | 1.0 |  |  |  | 1.0 |  |  |
| 1-2 | 5565(87.1) | 823(12.9) |  | 1.95 | 1.59-2.39 | <.001 |  | 1.74 | 1.41-2.16 | <.001 |
| 3-4 | 4157(78.4) | 1146(21.6) |  | 3.63 | 2.96-4.45 | <.001 |  | 2.88 | 2.32-3.57 | <.001 |
| 5-6 | 1048(67.4) | 508(32.6) |  | 6.38 | 5.12-7.96 | <.001 |  | 4.78 | 3.76-6.07 | <.001 |
| **SS** |  |  |  |  |  |  |  |  |  |  |
| high | 3251(89.4) | 384(10.6) |  | 1.0 |  |  |  | 1.0 |  |  |
| moderate | 6107(84.0) | 1165(16.0) |  | 1.62 | 1.43-1.83 | <.001 |  | 1.44 | 1.26-1.64 | <.001 |
| low | 2874(73.4) | 1039(26.6) |  | 3.06 | 2.70-3.48 | <.001 |  | 2.04 | 1.77-2.35 | <.001 |
| **SA** |  |  |  |  |  |  |  |  |  |  |
| **ACEs** |  |  |  |  |  |  |  |  |  |  |
| 0 | 1547(98.3) | 26(1.7) |  | 1.0 |  |  |  | 1.0 |  |  |
| 1-2 | 6225(97.4) | 163(2.6) |  | 1.56 | 1.03-2.37 | .037 |  | 1.37 | 0.90-2.10 | .147 |
| 3-4 | 5011(94.5) | 292(5.5) |  | 3.47 | 2.31-5.20 | <.001 |  | 2.60 | 1.71-3.97 | <.001 |
| 5-6 | 1388(89.2) | 168(10.8) |  | 7.20 | 4.74-10.95 | <.001 |  | 5.01 | 3.21-7.82 | <.001 |
| **SS** |  |  |  |  |  |  |  |  |  |  |
| high | 3534(97.2) | 101(2.8) |  | 1.0 |  |  |  | 1.0 |  |  |
| moderate | 7032(96.7) | 240(3.3) |  | 1.19 | .94-1.51 | .140 |  | 1.08 | 0.85-1.39 | .525 |
| low | 3605(92.1) | 308(7.9) |  | 2.99 | 2.38-3.76 | <.001 |  | 1.94 | 1.51-2.49 | <.001 |

Abbreviations: SI= suicidal ideation; SA= suicidal attempt; ACEs=Adverse childhood experiences; SS=social support

^a^ Unadjusted model

^b^ Adjusted for gender, age, regional areas, school, urban/rurality, mother’s education level, economic status of family, psychological symptoms, ACEs and SS

**Table SD3. Number, % and OR of NSSI (≥1)** by score of ACEs and level of SS in girls and boys, and their gender ratio

| Variable | Girls | | | |  | Boys | | | |  | Ratio of two ORs in girls vs boys | |
| --- | --- | --- | --- | --- | --- | --- | --- | --- | --- | --- | --- | --- |
|  | n(%) |  | OR(95%CI) ^+^ | *P* |  | n(%) |  | OR(95%CI) ^+^ | *P* |  | ROR | *P*-Value ^*^ |
| **ACEs** |  |  |  |  |  |  |  |  |  |  |  |  |
| 0 | 152(17.2) |  | 1.0 |  |  | 157(22.7) |  | 1.0 |  |  |  |  |
| 1-2 | 1039(31.3) |  | 1.88(1.55-2.30) | <.001 |  | 1039(33.8) |  | 1.64(1.34-2.00) | <.001 |  | 1.15 | .171 |
| 3-4 | 1181(45.4) |  | 2.87(2.33-3.53) | <.001 |  | 1238(45.8) |  | 2.48(2.01-3.05) | <.001 |  | 1.16 | .165 |
| 5-6 | 390(60.7) |  | 5.17(3.99-6.68) | <.001 |  | 537(58.8) |  | 3.93(3.09-5.01) | <.001 |  | 1.32 | .064 |
| **SS** |  |  |  |  |  |  |  |  |  |  |  |  |
| high | 436(22.5) |  | 1.0 |  |  | 459(27.0) |  | 1.0 |  |  |  |  |
| moderate | 1441(37.8) |  | 1.65(1.45-1.89) | <.001 |  | 1419(41.1) |  | 1.51(1.32-1.73) | <.001 |  | 1.09 | .179 |
| low | 885(52.3) |  | 2.16(1.84-2.53) | <.001 |  | 1093(49.2) |  | 1.65(1.42-1.91) | <.001 |  | **1.33** | **.005** |

Abbreviations: NSSI=non-suicidal self-injury; ACEs=Adverse childhood experiences; SS=social support

^+^ Adjusted for age, regional areas, school, urban/rurality, mother’s education level, economic status of family, psychological symptoms, ACEs and SS

^*^ one-side *P* value

**Table SD4. Number, % and OR of NSSI (≥5)** by score of ACEs and level of SS in girls and boys, and their gender ratio

| Variable | Girls | | | |  | Boys | | | |  | Ratio of two ORs in girls vs boys | |
| --- | --- | --- | --- | --- | --- | --- | --- | --- | --- | --- | --- | --- |
|  | n(%) |  | OR(95%CI) ^+^ | *P* |  | n(%) |  | OR(95%CI) ^+^ | *P* |  | ROR | *P*-Value ^*^ |
| **ACEs** |  |  |  |  |  |  |  |  |  |  |  |  |
| 0 | 48(5.4) |  | 1.0 |  |  | 67(9.7) |  | 1.0 |  |  |  |  |
| 1-2 | 432(13.0) |  | 2.13(1.55-2.93) | <.001 |  | 489(15.9) |  | 1.62(1.22-2.15) | .001 |  | 1.31 | .104 |
| 3-4 | 587(22.6) |  | 3.25(2.36-4.49) | <.001 |  | 662(24.5) |  | 2.43(1.83-3.23) | <.001 |  | 1.34 | .089 |
| 5-6 | 229(35.6) |  | 5.67(3.96-8.11) | <.001 |  | 342(37.5) |  | 4.06(2.97-5.55) | <.001 |  | 1.40 | .084 |
| **SS** |  |  |  |  |  |  |  |  |  |  |  |  |
| high | 161(8.3) |  | 1.0 |  |  | 205(12.1) |  | 1.0 |  |  |  |  |
| moderate | 637(16.7) |  | 1.67(1.37-2.02) | <.001 |  | 692(20.0) |  | 1.55(1.29-1.85) | <.001 |  | 1.08 | .291 |
| low | 498(29.4) |  | 2.32(1.88-2.87) | <.001 |  | 663(29.9) |  | 1.90(1.58-2.30) | <.001 |  | 1.22 | .083 |

Abbreviations: NSSI=non-suicidal self-injury; ACEs=Adverse childhood experiences; SS=social support

^+^ Adjusted for age, regional areas, school, urban/rurality, mother’s education level, economic status of family, psychological symptoms, ACEs and SS

^*^ one-side *P* value

**Table DS5.** Number, % and OR of SI by score of ACEs and level of SS in girls and boys, and their gender ratio

| Variables | Girls | | | |  | Boys | | | |  | Ratio of two ORs in girls vs boys ^c^ | |
| --- | --- | --- | --- | --- | --- | --- | --- | --- | --- | --- | --- | --- |
|  | n(%) | OR(95%CI) ^a^ | OR(95%CI) ^b^ | *P*-Value ^b^ |  | n(%) | OR(95%CI) ^a^ | OR(95%CI) ^b^ | *P*-Value ^b^ |  | ROR | *P*-Value ^*^ |
| **ACEs** |  |  |  |  |  |  |  |  |  |  |  |  |
| 0 | 66(7.5) | 1.0 | 1.0 |  |  | 45(6.5) | 1.0 | 1.0 |  |  |  |  |
| 1-2 | 503(15.2) | 2.21 (1.69-2.89) | 1.92(1.46-2.54) | <.001 |  | 320(10.4) | 1.67(1.21-2.31) | 1.49 (1.07-2.09) | .020 |  | 1.29 | .126 |
| 3-4 | 654(25.1) | 4.15(3.18-5.42) | 3.12(2.35-4.14) | <.001 |  | 492(18.2) | 3.20(2.33-4.39) | 2.56 (1.83-3.59) | <.001 |  | 1.22 | .189 |
| 5-6 | 242(37.6) | 7.46(5.54-10.04) | 5.10(3.69-7.04) | <.001 |  | 266(29.1) | 5.90(4.23-8.24) | 4.32(3.01-6.22) | <.001 |  | 1.18 | .252 |
| **SS** |  |  |  |  |  |  |  |  |  |  |  |  |
| high | 227(11.7) | 1.0 | 1.0 |  |  | 157(9.2) | 1.0 | 1.0 |  |  |  |  |
| moderate | 700(18.3) | 1.69(1.44-1.98) | 1.45(1.22-1.72) | <.001 |  | 465(13.5) | 1.53(1.26-1.85) | 1.42(1.16-1.75) | .001 |  | 1.02 | .439 |
| low | 538(31.8) | 3.50(2.95-4.16) | 2.11(1.74-2.56) | <.001 |  | 501(22.6) | 2.87(2.37-3.47) | 1.97(1.59-2.43) | <.001 |  | 1.07 | .319 |

Abbreviations: SI=suicidal ideation; ACEs=Adverse childhood experiences; SS=social support

^a^ Unadjusted model

^b^ Adjusted for age, regional areas, school, urban/rurality, mother’s education level, economic status of family, psychological symptoms, ACEs and SS

^c^ Calculated by adjusted OR

^*^ one-side *P* value

**Table DS6. Number, % and OR of NSSI (≥3)** in each of ACEs categories in girls and boys, and their gender ratio

| Variables | Girls | | | |  | Boys | | | |  | Ratio of two ORs in girls vs boys ^c^ | |
| --- | --- | --- | --- | --- | --- | --- | --- | --- | --- | --- | --- | --- |
|  | n(%) | OR(95%CI) ^a^ | OR(95%CI) ^b^ | *P*-Value ^b^ |  | n(%) | OR(95%CI) ^a^ | OR(95%CI) ^b^ | *P*-Value ^b^ |  | ROR | *P*-Value ^*^ |
| **Emotional abuse** |  |  |  |  |  |  |  |  |  |  |  |  |
| no | 581(15.4) | 1.0 | 1.0 |  |  | 931(21.8) | 1.0 | 1.0 |  |  |  |  |
| yes | 1230(33.6) | 2.78(2.49-3.11) | 2.20(1.94-2.49) | <.001 |  | 1130(36.4) | 2.05(1.85-2.27) | 1.78(1.59-2.00) | <.001 |  | **1.24** | **.007** |
| **Physical abuse** |  |  |  |  |  |  |  |  |  |  |  |  |
| no | 1332(21.5) | 1.0 | 1.0 |  |  | 1399(24.9) | 1.0 | 1.0 |  |  |  |  |
| yes | 479(38.5) | 2.28(2.01-2.60) | 1.79(1.55-2.07) | <.001 |  | 662(37.5) | 1.81(1.62-2.03) | 1.64(1.45-1.86) | <.001 |  | 1.09 | .184 |
| **Sexual abuse** |  |  |  |  |  |  |  |  |  |  |  |  |
| no | 1554(23.1) | 1.0 | 1.0 |  |  | 1564(25.6) | 1.0 | 1.0 |  |  |  |  |
| yes | 257(36.2) | 1.90(1.61-2.23) | 1.69(1.41-2.02) | <.001 |  | 497(39.4) | 1.90(1.67-2.15) | 1.64(1.43-1.89) | <.001 |  | 1.03 | .398 |
| **Emotional neglect** |  |  |  |  |  |  |  |  |  |  |  |  |
| no | 536(18.8) | 1.0 | 1.0 |  |  | 566(23.0) | 1.0 | 1.0 |  |  |  |  |
| yes | 1275(27.7) | 1.65(1.47-1.85) | 1.30(1.14-1.47) | <.001 |  | 1495(30.4) | 1.46(1.31-1.64) | 1.27(1.12-1.43) | <.001 |  | 1.02 | .398 |
| **Physical neglect** |  |  |  |  |  |  |  |  |  |  |  |  |
| no | 700(20.7) | 1.0 | 1.0 |  |  | 688(24.9) | 1.0 | 1.0 |  |  |  |  |
| yes | 1111(27.4) | 1.44(1.30-1.61) | 1.20(1.06-1.35) | .004 |  | 1373(29.8) | 1.28(1.15-1.43) | 1.16(1.04-1.31) | .002 |  | 1.03 | .345 |
| **Household dysfunction** |  |  |  |  |  |  |  |  |  |  |  |  |
| no | 781(18.0) | 1.0 | 1.0 |  |  | 887(21.2) | 1.0 | 1.0 |  |  |  |  |
| yes | 1030(33.2) | 2.27(2.04-2.53) | 1.77(1.57-1.99) | <.001 |  | 1174(36.7) | 2.15(1.94-2.38) | 1.67(1.49-1.87) | <.001 |  | 1.06 | .244 |

Abbreviations: NSSI=non-suicidal self-injury; ACEs=Adverse childhood experiences

^a^ Unadjusted model

^b^ Adjusted for age, regional areas, school, urban/rurality, mother’s education level, economic status of family, psychological symptoms and social support.

^c^ Calculated by adjusted OR

^*^one-side *P* value

**Table DS7.** Number, % and OR of SI in each of ACEs categories in girls and boys, and their gender ratio

| Variables | Girls | | | |  | Boys | | | |  | Ratio of two ORs in girls vs boys ^c^ | |
| --- | --- | --- | --- | --- | --- | --- | --- | --- | --- | --- | --- | --- |
|  | n(%) | OR(95%CI) ^a^ | OR(95%CI) ^b^ | *P*-Value ^b^ |  | n(%) | OR(95%CI) ^a^ | OR(95%CI) ^b^ | *P*-Value ^b^ |  | ROR | *P*-Value ^*^ |
| **Emotional abuse** |  |  |  |  |  |  |  |  |  |  |  |  |
| no | 446(11.8) | 1.0 | 1.0 |  |  | 438(10.3) | 1.0 | 1.0 |  |  |  |  |
| yes | 1019(27.8) | 2.88(2.55-3.25) | 2.33(2.04-2.67) | <.001 |  | 685(22.1) | 2.48(2.17-2.82) | 2.17(1.88-2.50) | <.001 |  | 1.07 | .238 |
| **Physical abuse** |  |  |  |  |  |  |  |  |  |  |  |  |
| no | 1056(17.0) | 1.0 | 1.0 |  |  | 717(12.8) | 1.0 | 1.0 |  |  |  |  |
| yes | 409(32.9) | 2.38(2.08-2.73) | 1.84(1.59-2.14) | <.001 |  | 406(23.0) | 2.04(1.78-2.34) | 1.76(1.52-2.05) | <.001 |  | 1.05 | .340 |
| **Sexual abuse** |  |  |  |  |  |  |  |  |  |  |  |  |
| no | 1270(18.9) | 1.0 | 1.0 |  |  | 856(14.0) | 1.0 | 1.0 |  |  |  |  |
| yes | 195(27.5) | 1.63(1.37-1.95) | 1.39(1.14-1.68) | .001 |  | 267(21.2) | 1.65(1.42-1.93) | 1.36(1.14-1.61) | <.001 |  | 1.02 | .435 |
| **Emotional neglect** |  |  |  |  |  |  |  |  |  |  |  |  |
| no | 415(14.6) | 1.0 | 1.0 |  |  | 242(9.8) | 1.0 | 1.0 |  |  |  |  |
| yes | 1050(22.8) | 1.73(1.53-1.96) | 1.44(1.25-1.65) | <.001 |  | 881(17.9) | 2.00(1.72-2.33) | 1.73(1.47-2.03) | <.001 |  | **0.83** | **.046** |
| **Physical neglect** |  |  |  |  |  |  |  |  |  |  |  |  |
| no | 576(17.0) | 1.0 | 1.0 |  |  | 335(12.1) | 1.0 | 1.0 |  |  |  |  |
| yes | 889(21.9) | 1.37(1.22-1.53) | 1.21(1.06-1.38) | .005 |  | 788(17.1) | 1.50(1.31-1.72) | 1.33(1.15-1.55) | <.001 |  | 0.91 | .176 |
| **Household dysfunction** |  |  |  |  |  |  |  |  |  |  |  |  |
| no | 671(15.5) | 1.0 | 1.0 |  |  | 482(11.5) | 1.0 | 1.0 |  |  |  |  |
| yes | 794(25.6) | 1.88(1.68-2.11) | 1.65(1.45-1.88) | <.001 |  | 641(20.0) | 1.92(1.69-2.18) | 1.62(1.41-1.87) | <.001 |  | 1.02 | .426 |

Abbreviations: SI=suicidal ideation; ACEs=Adverse childhood experiences

^a^ Unadjusted model

^b^ Adjusted for age, regional areas, school, urban/rurality, mother’s education level, economic status of family, psychological symptoms and social support.

^c^ Calculated by adjusted OR

^*^one-side *P* value

**Table DS8.** Number, % and OR of SA in each of ACEs categories in girls and boys, and their gender ratio

| Variables | Girls | | | |  | Boys | | | |  | Ratio of two ORs in girls vs boys ^c^ | |
| --- | --- | --- | --- | --- | --- | --- | --- | --- | --- | --- | --- | --- |
|  | n(%) | OR(95%CI) ^a^ | OR(95%CI) ^b^ | *P*-Value ^b^ |  | n(%) | OR(95%CI) ^a^ | OR(95%CI) ^b^ | *P*-Value ^b^ |  | ROR | *P*-Value ^*^ |
| **Emotional abuse** |  |  |  |  |  |  |  |  |  |  |  |  |
| no | 95(2.5) | 1.0 | 1.0 |  |  | 99(2.3) | 1.0 | 1.0 |  |  |  |  |
| yes | 295(8.1) | 3.40(2.68-4.30) | 2.65(2.06-3.41) | <.001 |  | 160(5.2) | 2.29(1.77-2.95) | 1.95(1.48-2.57) | <.001 |  | 1.36 | .054 |
| **Physical abuse** |  |  |  |  |  |  |  |  |  |  |  |  |
| no | 237(3.8) | 1.0 | 1.0 |  |  | 157(2.8) | 1.0 | 1.0 |  |  |  |  |
| yes | 153(12.3) | 3.52(2.85-4.36) | 2.62(2.08-3.30) | <.001 |  | 102(5.8) | 2.13(1.65-2.76) | 1.73(1.32-2.28) | <.001 |  | **1.51** | **.011** |
| **Sexual abuse** |  |  |  |  |  |  |  |  |  |  |  |  |
| no | 320(4.8) | 1.0 | 1.0 |  |  | 181(3.0) | 1.0 | 1.0 |  |  |  |  |
| yes | 70(9.9) | 2.20(1.67-2.88) | 1.80(1.35-2.41) | <.001 |  | 78(6.2) | 2.16(1.65-2.84) | 1.79(1.33-2.41) | <.001 |  | 1.01 | .490 |
| **Emotional neglect** |  |  |  |  |  |  |  |  |  |  |  |  |
| no | 91(3.2) | 1.0 | 1.0 |  |  | 63(2.6) | 1.0 | 1.0 |  |  |  |  |
| yes | 299(6.5) | 2.10(1.66-2.67) | 1.56(1.21-2.01) | .001 |  | 196(4.0) | 1.58(1.19-2.11) | 1.26(0.93-1.71) | .130 |  | 1.20 | .177 |
| **Physical neglect** |  |  |  |  |  |  |  |  |  |  |  |  |
| no | 122(3.6) | 1.0 | 1.0 |  |  | 75(2.7) | 1.0 | 1.0 |  |  |  |  |
| yes | 268(6.6) | 1.89(1.52-2.35) | 1.58(1.25-2.00) | <.001 |  | 184(4.0) | 1.49(1.14-1.96) | 1.25(0.93-1.67) | .134 |  | 1.23 | .137 |
| **Household dysfunction** |  |  |  |  |  |  |  |  |  |  |  |  |
| no | 165(3.8) | 1.0 | 1.0 |  |  | 102(2.4) | 1.0 | 1.0 |  |  |  |  |
| yes | 225(7.3) | 1.98(1.61-2.44) | 1.67(1.33-2.09) | <.001 |  | 157(4.9) | 2.06(1.60-2.65) | 1.75(1.33-2.30) | <.001 |  | 0.89 | .257 |

Abbreviations: SA= suicidal attempt; ACEs=Adverse childhood experiences

^a^ Unadjusted model

^b^ Adjusted for age, regional areas, school, urban/rurality, mother’s education level, economic status of family, psychological symptoms and social support.

^c^ Calculated by adjusted OR

^*^one-side *P* value

**Table DS9** The prevalence of SS level by score of ACEs, n(%)

| **ACEs** | **SS** | | | *p*-Value* |
| --- | --- | --- | --- | --- |
|  | high | moderate | low |  |
| 0 | 668(42.5) | 771(49.0) | 134(8.5) | <.001 |
| 1-2 | 1892(29.6) | 3239(50.7) | 1257(19.7) |  |
| 3-4 | 893(16.8) | 2559(48.3) | 1851(34.9) |  |
| 5-6 | 182(11.7) | 703(45.2) | 671(43.1) |  |

Abbreviations: ACEs=Adverse childhood experiences; SS=social support

*trend p value
